# Akebia Saponin D prevents axonal loss against TNF-induced optic nerve damage with autophagy modulation

**DOI:** 10.1007/s11033-020-06008-y

**Published:** 2020-11-28

**Authors:** Kana Sase, Chihiro Tsukahara, Naoki Fujita, Ibuki Arizono, Hitoshi Takagi, Yasushi Kitaoka

**Affiliations:** 1 Department of Ophthalmology, St. Marianna University School of Medicine, Kawasaki, Kanagawa Japan; 2 Department of Molecular Neuroscience, St. Marianna University Graduate School of Medicine, 2-16-1 Sugao, Miyamae-ku, Kawasaki, Kanagawa 216-8511 Japan

**Keywords:** TNF, Optic nerve, Axon, p62, Autophagy, p38

## Abstract

Akebia Saponin D (ASD), a triterpenoid saponin, was shown to have protective effects in certain neuronal cells. The purpose of the present study was to investigate the possibility of ASD to prevent tumor necrosis factor (TNF)-induced axonal loss and the ASD modulation of the biologic process of autophagy in optic nerves. Rats were given intravitreal administration of TNF, simultaneous administration of 2, 20, or 200 pmol ASD and TNF, or ASD alone. LC3-II and p62 expression, which is a marker of autophagic flux, and phosphorylated p38 (p-p38) expression in optic nerves were examined by immunoblot analysis. Morphometric analysis revealed a significant ameliorated effect of ASD against TNF-induced optic nerve damage. p62 was significantly increased in the optic nerve in TNF-treated eyes, but this increase was totally prevented by ASD. The ASD alone injection showed significant reduction of p62 levels compared with the PBS-treated control eyes. LC3-II was significantly increased by ASD treatment in the TNF-injected eyes. p-p38 was significantly increased in the optic nerve in TNF-treated eyes, but this increase was completely prevented by ASD. The protective effects of ASD may be associated with enhanced autophagy activation and inhibition of p-p38.

## Introduction

Autophagy, a cellular process which includes the degradation of cytoplasmic and axoplasmic components, has been linked to the pathophysiology of certain human diseases, such as neurodegenerative diseases [1–4]. Several studies have demonstrated the relationship between autophagy and glaucoma, however, its role related to retinal ganglion cells (RGCs) and the optic nerve remains controversial [5–8]. Glaucoma is a neurodegenerative disease in which RGCs and their axons progressively degenerate. Though a protective role of autophagy has been reported in an experimental hypertensive glaucoma model [8], a detrimental role of autophagy has been reported in the hypertensive glaucoma model [9]. Our previous studies showed that enhanced autophagy flux is involved in axonal protection against in the hypertensive glaucoma model and tumor necrosis factor (TNF)-induced optic nerve degeneration model [10, 11].

Akebia saponin D (ASD) is extracted from herbal medicine *Dipsacus asper Wall*. A previous report showed that ASD increased autophagic flux in the mice hepatic steatosis model [12]. In neuronal cells, it was reported that ASD has a neuroprotective effect against cytotoxicity induced by amyloid-β in PC12 cells [13], and ASD has been shown to ameliorate not only Alzheimer’s disease-associated inflammation but also memory disorder [14]. In addition, ASD suppresses mitogen-activated protein kinase (MAPK) family phosphorylations, implying that this pathway may be linked to the neuroprotection mechanism of ASD [15]. Among MAPK, a previous study demonstrated a detrimental role of phosphorylated p38 (p-p38) in RGCs in an optic nerve crush model [16]. Notably, a recent study showed that inhibition of p38 resulted in enhanced autophagy induction in a spinal cord injury rat model [17], suggesting a relationship between p38 and autophagy. However, the role and localization of p-p38 in optic nerve damage remains unclear. In the current study, we investigated whether ASD prevents TNF-induced axonal loss, and modulates autophagy status and the p-p38 level in optic nerve.

## Material and methods

### Animals

Experiments were performed on 8-week-old male Wistar rats. All studies were conducted according to the Association for Research in Vision and Ophthalmology (ARVO) statement on the Use of Animals in Ophthalmic and Vision Research and were approved by the Ethics Committee of the Institute of Experimental Animals of St. Marianna University Graduate School of Medicine. The animals were maintained under controlled conditions, with temperature at 23 ± 1 °C, humidity at 55 ± 5%, and light from 06:00 to 18:00.

### Administration of TNF

Intravitreal injection was carried out as described previously [18]. In anesthetized rats, a single 2-μl injection of 10 ng TNF was administered intravitreally to the right eye. Phosphate-buffered saline (PBS) was used as a control. For the ASD administration, simultaneous injection of 2, 20, or 200 pmol of ASD (Sigma-Aldrich) and 10 ng of TNF was performed intravitreally. One or 2 weeks after the administration, the rats were euthanized and the eyes were removed.

### Immunoblot analysis

One and two weeks after administration, optic nerves (4 mm in length) were isolated, homogenized, and centrifuged at 15,000 × g for 15 min at 4 °C as described previously [19]. Supernatants were used for measurements of protein concentrations. Samples (3 μg per lane, but 6 μg per lane for p-p38) were applied to gels and transferred to membranes. After blocking, membranes were first reacted with anti-p62/SQSTM1 antibody (1:200; MBL), anti-microtube-associated protein light chain 3 (LC3) antibody (1:500; MBL), anti-p-p38 antibody (1:200; Cell Signaling), or anti-β-actin antibody (1:5000; Sigma-Aldrich). Then, membranes were exposed to secondary antibodies; anti-rabbit IgG antibody (1:5000; Cappel) or anti-mouse IgG antibody. Immunoblotting was comfirmed with an ECL detection system.

### Determine axon number

Quantification of each axon was conducted with optic nerves from 32 rats as described previously [18, 19]. The optic nerve specimens were obtained 2 weeks after intravitreal injection. They were immersed in Karnovsky’s solution overnight and acrylic resin blocks were made. Cross sections were made starting 1 mm from the sclera and reacted a solution of 1% paraphenylen-diamine (Sigma-Aldrich). Five images (center and periphery in quadrant; 1446.5 μm^2^ each; total area of 7232.3 μm^2^ per eye) were acquired and quantified using Aphelion image-processing software. The number of axons per eye was averaged and presented as the number/mm^2^.

### Immunohistochemistry of p-p38

The eyes were enucleated 1 week after injection and eye caps were made. The eye cap samples were immersed in 4% paraformaldehyde. Paraffinized sections were made longitudinally through the optic disc. The primary antibodies were anti-p-p38 antibody (1:100; Cell Signaling) and anti-neurofilament-L antibody (a marker of neurons; 1:100; DAKO). The secondary antibodies used were FITC-labeled and rhodamine-labeled antibodies. The samples on slides were mounted in DAPI-containing medium (Vector Laboratories, Inc., Burlingame, CA, USA).

### Statistical analysis

Data are presented as mean ± S.E.M. Differences among groups in axon data and immunoblot data were analyzed using one-way ANOVA, following by Dunnett’s post hoc test. A probability value of less than 0.05 was considered to represent a statistically significant difference.

## Results

### Axon counting

**Fig. 1:**
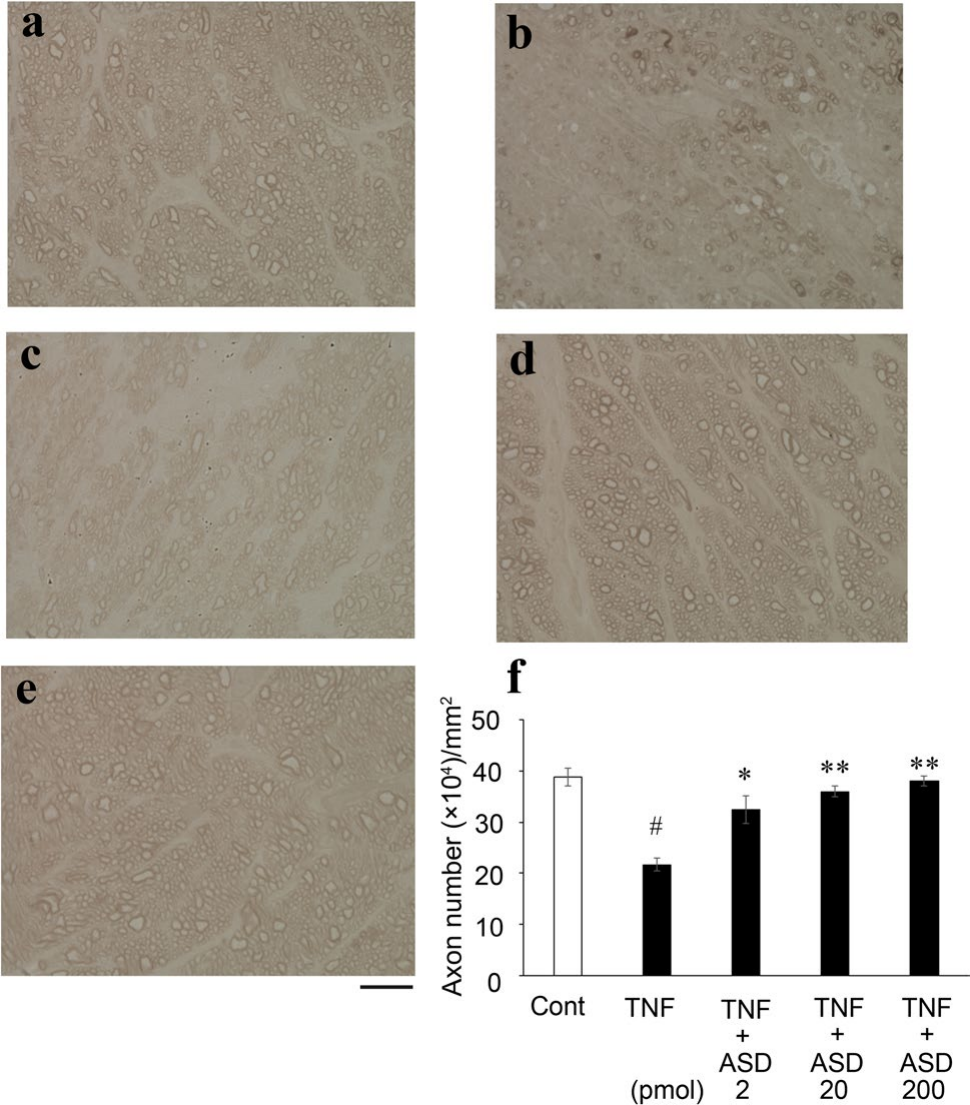
ASD suppresses axon loss induced by TNF. Cross-sectioned axons at 2 weeks after (**a**) PBS injection, (**b**) TNF injection, (**c**) TNF + 2 pmol ASD injection, (**d**) TNF + 20 pmol ASD injection, or (**e**) TNF + 200 pmol ASD injection. Scale: 10 μm; magnification, X100. (**f**) Number of axons. *n* = 6–7. ^#^*P* < 0.0001 compared with PBS; *P < 0.0005 compared with TNF; **P < 0.0001 compared with TNF

Consistent with our previous study [11], compared with the control group (Fig. 1a), the TNF group showed significant degenerative changes in the optic nerve (Fig. 1b). Compared with degenerative axons in the TNF group, the ASD group showed significant protective effects in the 2, 20, and 200 pmol-treated groups (Fig. 1c–e). The protective effect of ASD was significant and dose dependent. (Fig. 1f).

### Western blot of p62 in optic nerve

**Fig. 2:**
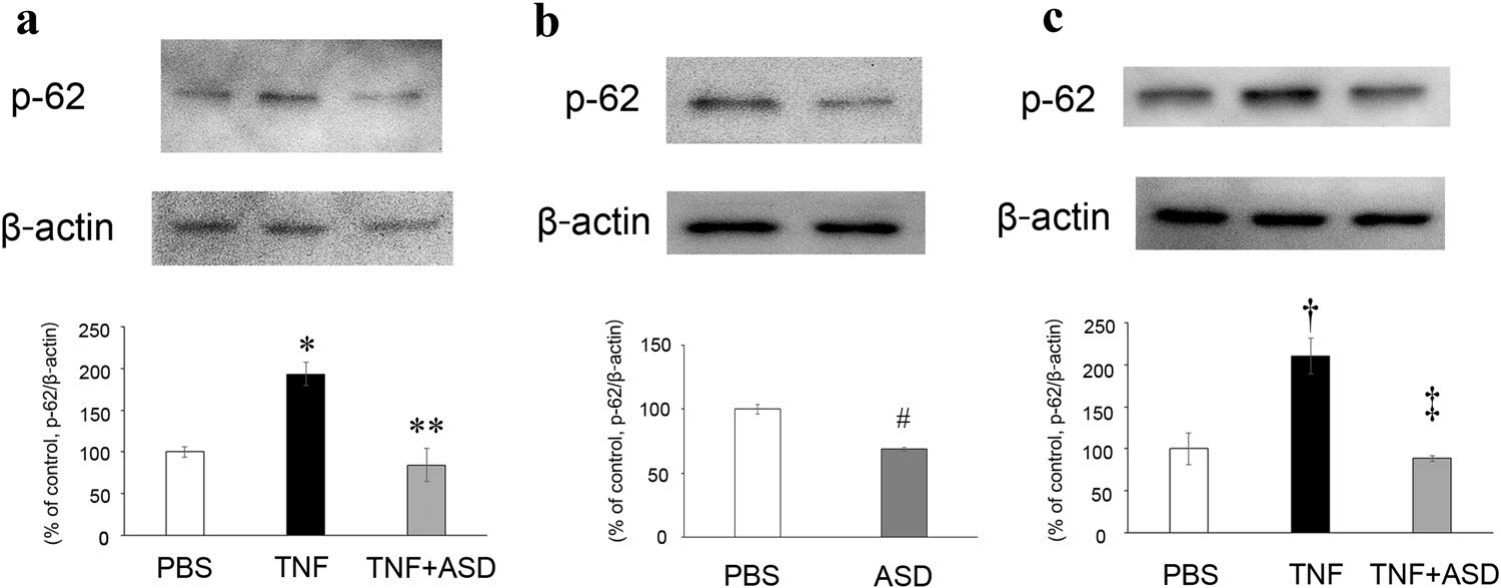
Effects of TNF and ASD on p62 expression changes in the optic nerves. One week after administration of PBS, TNF, or TNF + 200 pmol ASD (**a**) and PBS or 200 pmol ASD (**b**) *n* = 4. *P < 0.01 compared with PBS; **P < 0.005 compared with TNF; ^#^*P* < 0.001 compared with PBS. Two weeks after administration of PBS, TNF, or TNF + 200 pmol ASD (**c**) *n* = 4.^†^*P* < 0.05 compared with PBS, ^‡^*P* < 0.05 compared with TNF

Consistent with our previous study [11], the expression of p62 was significantly increased by TNF at 1 week (Fig. 2a). The ASD alone injection decreased the expression of p62 (Fig. 2b). Moreover, the TNF-increased p62 expression was significantly decreased by ASD at 1 week (Fig. 2a). Furthermore, the TNF-increased p62 expression was significantly decreased by ASD at 2 weeks (Fig. 2c).

### Western blot of LC3-II in optic nerve

**Fig. 3:**
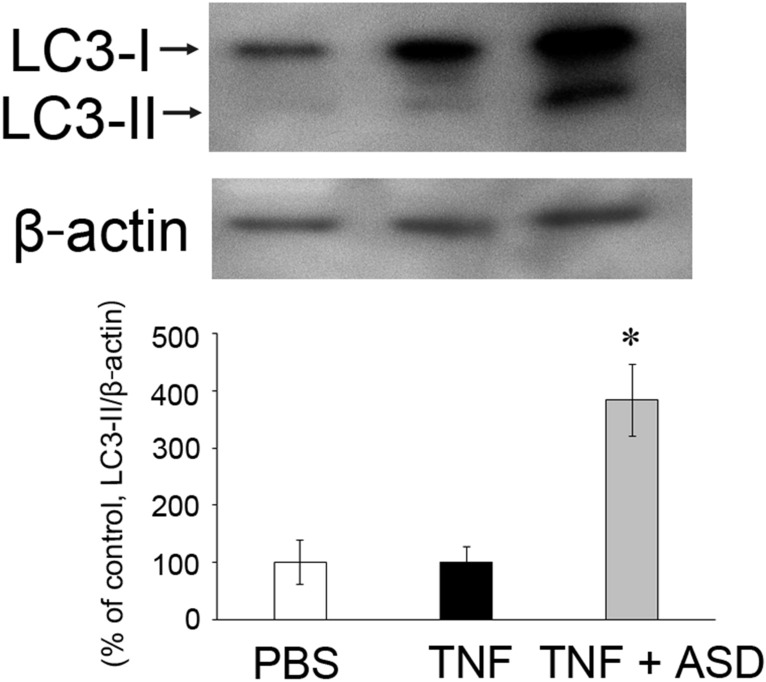
Effects of TNF and ASD on LC3-II expression changes in the optic nerves. Two weeks after administration. *n* = 3. *P < 0.05 compared with TNF

There was no significant change in the LC3-II level in optic nerve 2 weeks after TNF injection. The ASD injection significantly increased LC3-II level as compared with TNF alone (Fig. 3).

### Western blot of p-p38 in optic nerve

**Fig. 4:**
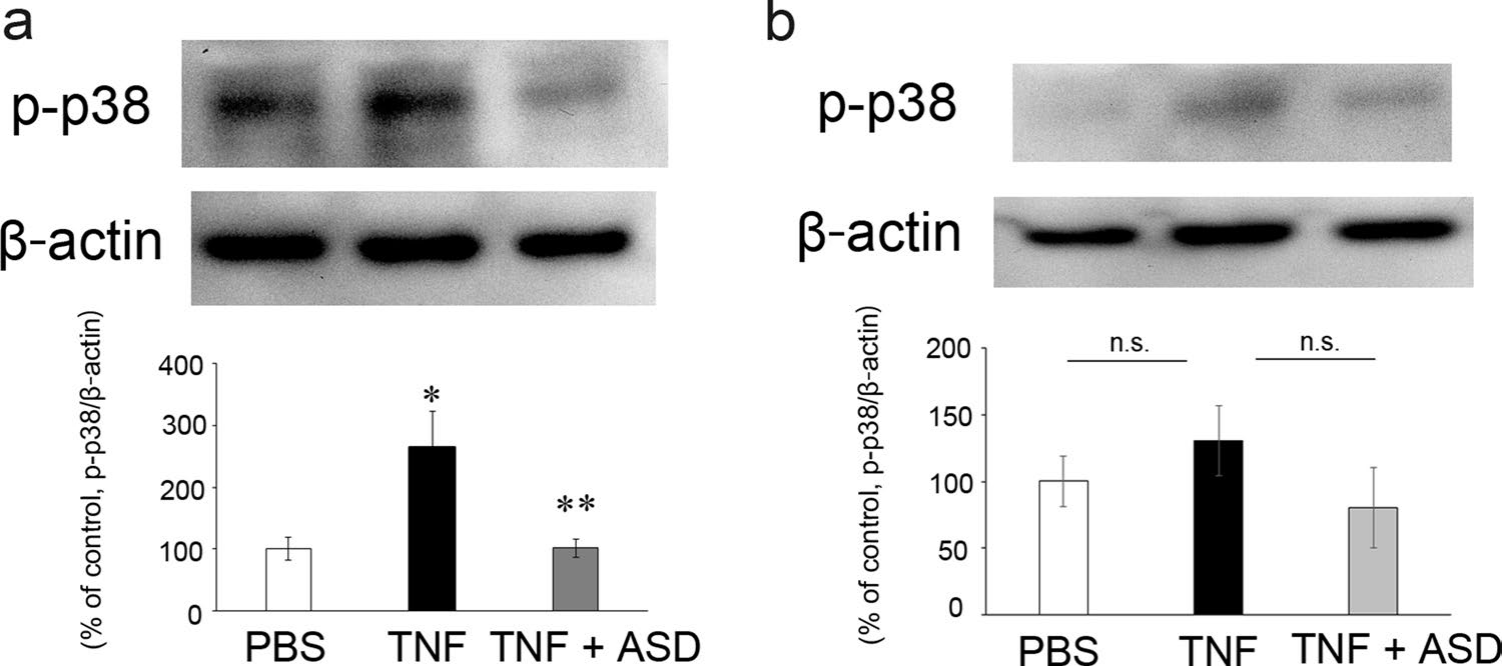
Effects of TNF and ASD on p-p38 expression changes in the optic nerves. One week after administration (**a**) *n* = 6–7. *P < 0.05 compared with PBS; **P < 0.05 compared with TNF. Two weeks after administration (**b**) *n* = 3

A previous study reported that p-p38 exists in RGCs [20], but no report in optic nerve. In this study, we investigated whether p-p38 exists in optic nerve. We confirmed the expression of p-p38 in the optic nerve. The p-p38 level was significantly increased by TNF at 1 week. Additionally, the TNF-increased p-p38 expression was significantly decreased by ASD at 1 week (Fig. 4a). We previously found that axonal loss started 1 week after TNF injection [18], and the molecular events at 1 week when before axon loss becomes obvious are important for clarifying the mechanism of axonal degeneration. At 2 weeks when axon loss becomes obvious, the p-p38 level tended to be increased by TNF, but this was not statistically significant (Fig. 4b). Also, ASD treatment decreased the TNF-induced increment, but this was not statistically significant (Fig. 4b).

### Localization of p-p38 in the optic nerve

**Fig. 5:**
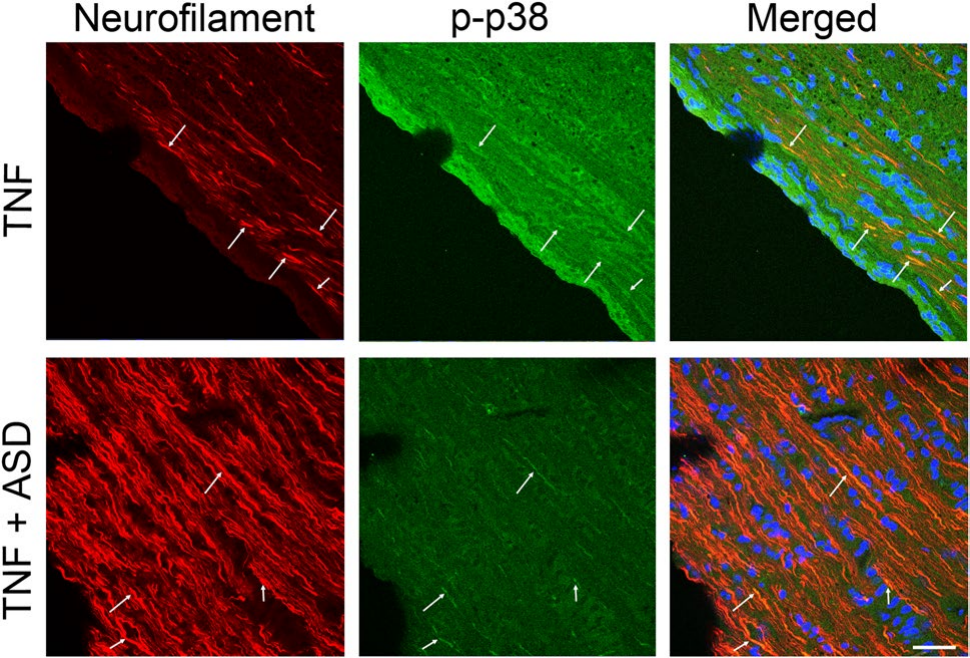
The double immunostaining of the optic nerve 1 week after administration. Co-localization of p-p38 with neurofilaments in the optic nerve in the TNF group (upper panel). In the TNF + ASD group (lower panel), expression of p-p38 seemed to be suppressed compared to the TNF group. Arrows indicate colocalizations. Scale: 50 μm

Immunohistochemical study found abundant p-p38 immunoreactivity in TNF-treated group and that some of these immunoreactivities were colocalized with neurofilament immunoreactivity in optic nerve (Fig. 5, upper panel). It seemed that the expression of p-p38 was modest in the TNF + ASD group compared to the TNF group (Fig. 5, lower panel). These findings suggested that p-p38 is present in the optic nerve axon, and the expression may be decreased by ASD.

## Discussion

Our previous study showed that induction of autophagy is involved in axonal protection against TNF- induced degeneration in the optic nerve [11, 21]. Although p62 facilitates substrate-specific degradation through the ubiquitin-proteasome system [22], it is a multifunctional protein that interacts with a central component of the autophagy machinery and decreased p62 levels are associated with autophagy activation [23]. In addition, increases in the level of LC3-II are not measures of autophagic flux per se but these increases can reflect the induction of the autophagosome or inhibition of autophagosome clearance [23]. In the current study, ASD which is known to be an autophagy inducer, exerted significant axonal protection in TNF-induced axonal degeneration. Because inhibition of autophagy was shown to correlate with increased levels of p62 [23], increased levels of p62 may correlate with inhibition of autophagy in TNF-induced optic nerve degeneration, although there was no significant changes in LC3-II in optic nerve after TNF injection. ASD upregulated LC3-II and decreased p62 levels, indicating that ASD activated autophagy. Therefore, the current study also demonstrated that autophagy is involved in axonal protection by ASD in the optic nerve. MAPK is known to respond to various stresses. In Alzheimer’s disease mouse and cell models, inhibition of p38 enhanced autophagy and significantly decreased amyloid β [24]. p38 expression was increased after subarachnoid hemorrhage (SAH) that leads to mitochondria dysfunction due to abnormal autophagy [25]. Furthermore, p38 inhibitor was shown to reverse abnormal autophagy flux in a SAH model [25]. In the eye, it has been reported that p38 is activated in RGC by NMDA injection and optic nerve injury [16, 20]. Those studies also showed that inhibition of p38 protected RGCs death induced by NMDA and optic nerve injury [16, 20]. In the present study, p-p38 was activated by TNF in the optic nerve, and ASD reduced this activation. The association of autophagy and p38 is unclear, however, it is reported that p38 induced mTOR activation in the liver [26] and that p38 activated mTOR in cardiomyocytes [27]. Other reports also showed that p38 activated mTOR following oxidative stress in the human lung cancer cell line [28]. Because it is well known that mTOR suppresses autophagy, activated p38 suppresses autophagy. In the present study, the upregulated p-p38 level, induced by TNF, seemed to activate mTOR which leads to p62 accumulation. The peak of p-p38 elevation was 1 week after TNF injection, whereas significant elevations of p62 were observed at both 1 and 2 weeks, implicating that p-p38 exists upstream of p62. Treatment with ASD can inhibit this pathway, thereby exerting axonal protection.

In conclusion, these results suggested that the axonal protection of ASD may be involved in autophagy activation by inhibition of p-p38.
